# Piloting a language translation device for Mandarin-speaking patients presenting for radiotherapy treatment—assessing patient and radiation therapist perspectives

**DOI:** 10.1007/s00520-024-08438-x

**Published:** 2024-03-19

**Authors:** Darren Hunter, Nigel Anderson, Richard Oates, David Kok, Daniel Sapkaroski, Nicola Treffry, Caroline Wright

**Affiliations:** 1 Department of Medical Imaging and Radiation Sciences, School of Primary and Allied Health Care, Monash University, Clayton, VIC Australia; 2 Radiation Oncology, Olivia Newton-John Cancer Wellness & Research Centre, Austin Health, Heidelberg, VIC Australia; 3 Radiation Therapy Services, Peter MacCallum Cancer Centre, Bendigo, VIC Australia; 4 Department of Radiation Oncology, Peter MacCallum Cancer Centre, Moorabbin, VIC Australia; 5 Radiation Therapy Services, Peter MacCallum Cancer Centre, Parkville, VIC Australia

**Keywords:** Radiotherapy, Translation, CALD, Communication, Cancer, Patient care

## Abstract

**Purpose:**

Culturally and linguistically diverse (CALD) cancer patients report unmet informational and emotional needs when receiving radiotherapy (RT). This feasibility study aimed to evaluate the clinical use of an instant translation device (ITD) to facilitate communication between Mandarin-speaking patients and radiation therapists (RTTs) within the Australian public RT setting. The primary aim was to assess the ability to convey information relating to daily patient care and build rapport using the device.

**Methods:**

A single-arm prospective interventional trial was employed with patient and RTT participants. Eligible patient participants were aged 18 years or older, diagnosed with cancer, referred for RT with self-reported Mandarin as the primary language spoken at home. Patients who had previously received RT were excluded. Consenting patient participants completed a baseline assessment of health literacy (REALM-SF) and English proficiency (LexTALE). Surveys were administered to patients and consenting RTTs at the cessation of treatment, forming two distinct participant groups. Descriptive statistics were used to compare participant groups.

**Results:**

Eleven patients and 36 RTTs were recruited to the study. Descriptive statistics demonstrated participant group agreement in conveying treatment instructions, though differing experiences were reported against general conversation. Although the reporting of technical difficulties was inconsistent, both groups recommended the application of the ITD within the RT domain.

**Conclusion:**

This feasibility study demonstrated encouraging accounts of patients and RTTs with regard to ITD use in the context of RT treatment. Expanded, multi-institutional recruitment is required to yield statistical significance, inform the impact of the device, and determine requisite training requirements.

**Trial registration:**

HREC reference number: LNR/18/PMCC/115 (18/100L). HREC approval date: 10 July 2018.

**Supplementary Information:**

The online version contains supplementary material available at 10.1007/s00520-024-08438-x.

## Introduction

Radiation therapists (RTTs) play an integral role in supporting the emotional and informational needs of cancer patients [1, 2]. Clear and consistent communication between RTTs and cancer patients ensures patients feel safe, understood, engaged, educated, and informed [1, 2]. Employing a patient-centered approach to communication within the RT context can alleviate feelings of loneliness, distress, anxiety, and uncertainty [1, 2]. Moreover, this fosters parity in the patient-therapist relationship, allowing patients to build rapport, trust, and confidence in the RTTs [2].

Despite reported variation in information provision for RT patients broadly, literature suggests that culturally and linguistically diverse (CALD) patients are profoundly disadvantaged with unmet informational needs and are often omitted from global patient satisfaction surveys [3, 4]. This is evidenced by the 2018 Australian Health Literacy Survey, which demonstrated lower social support for healthcare needs among Australians who spoke a language other than English at home (19%, compared with 26% of English-speaking Australians) [5]. As such, in order to facilitate a culturally safe environment, RTTs should engage in communication, build trust, increase cultural awareness, and respond to the variable cultural needs of the community [6].

Nearly half of all people living in the state of Victoria, Australia (hereafter ‘Victorian’) are first-or-second-generation migrants; thus, the need for culturally appropriate health service provision is ever present within the community [7]. Among the 263 reported non-English languages spoken by Victorians, the most common was observed to be Mandarin (3.4%) [8]. The Victorian Language Services Guidelines provide legislative framework and indications for best practice where language translation/interpretation is required across public service industries, including healthcare. However, uptake and utility within the health context is variable [9, 10]. In a bid to overcome barriers of cost and access to professional interpreter services, research suggests that modern technology may offer some respite and resolution [4].

This feasibility study builds upon the validation study conducted in 2018 that demonstrated favorable English-to-Mandarin translation for common RTT phrases, using an instant translation device (ITD) [11].

The study aim was to evaluate the clinical use of an ITD to facilitate communication between Mandarin-speaking patients and RTTs within the Australian public RT setting. Thus, the primary objective of this research was to assess the clinical application of an ITD—specifically the ability to build rapport and convey information relating to daily patient care and treatment provided by RTTs. The secondary objectives of this study were as follows:

1. to evaluate the suitability and acceptance of ITD use within daily RT service provision—as determined by consenting patient and RTT participants, and
2. to establish the feasibility for scaled study across multiple RT departments.

## Methods

This multi-site feasibility study was developed as a single-arm prospective interventional trial, with a target recruitment of twenty patients over an 18-month period. Similar studies by Egestad [1] and Choi et al. [4] recruited eleven and nineteen participants, respectively—thus, the target recruitment was deemed suitable to gain robust data to inform feasibility.

Institutional and academic human research ethics committee (HREC) approval was granted in August 2018, with protocol amendments authorised in October 2019 (Monash University Ref 16,793; Peter MacCallum Cancer Centre Ref 40756). Site lead RTTs were appointed, undertook in-depth training, and had a thorough understanding of the study—including the inclusion/exclusion criteria. General training regarding the study and use of the ITD was provided to all RTTs across the participating service locations.

### Equipment

This study employed the use of the ITD to facilitate daily communication between participating patients and RTTs. The handheld device, Travis Touch Go (Travis®, Rotterdam, Netherlands), permits bi-directional communication, with a touch-screen interface. Prior validation testing deemed the device output viable for radiotherapy application in the context of English and Mandarin interpretation [11].

The ITD was used for engagement throughout daily RT treatment—including, but not limited to, general conversation, patient identification and treatment instructions. Use of the ITD was contained to the RTT and patient participant, exclusively. A device was housed at each treatment machine for use with any/all participants consenting to the trial. This ensured the device was charged, present for treatment and free from tampering/software updates.

### Sampling, recruitment, and inclusion/exclusion criteria

Screening of potential patient and RTT participants was conducted with use of the MOSAIQ® (version 1.6, ElektaAB, Stockholm, Sweden) record and verify system, with an electronic enrolment log recorded.

#### Patient participants

A referral to language services (Mandarin) for the initial radiation oncologist appointment served as a flag for possible eligibility. At RT simulation, eligible patients were explained the study aims, requirements, and rationale by site lead RTTs and provided a demonstration of the ITD, often with the support of an interpreter. This information was also provided in the patient information and consent form—translated into Chinese Simplified. RTTs advised that participation was voluntary and patients reserved the right to withdraw at any given time. Informed consent was obtained and patient participants were assigned a unique study identification number to track participation and late withdrawal. All data was otherwise de-identified.

Eligible patient participants were aged 18 years or older, diagnosed with cancer, referred for RT, and self-reported Mandarin as the primary language spoken at home. Patients who had previously received RT treatment were excluded from this research.

Patient recruitment commenced at two participating Victorian metropolitan public RT services in December 2018, expanding to two additional metropolitan locations and one regional centre in January 2020. At the onset of COVID-19 restrictions across Victoria, the investigators elected to close recruitment on 31 March 2020 with 18 participants (90%) successfully recruited. The investigators determined that this would satisfy analysis and mitigate the confounding effects of the COVID-19 patient experience.

#### RTT participants

Eligible RTT participants were identified as having delivered one or more fractions (treatments) of RT to any patient participant that was enrolled in the study. RTT participants, in completing the survey, provided implied consent. Participants were assigned a unique study identification number and de-identified by any demographical feature aside from the treatment site in which they worked. No further exclusion criteria applied.

Following the initial COVID-19 lockdown period, a staff survey was administered over three weeks in July–August 2020, thus completing data collection. Due to one service location failing to recruit to the study (Metro Site D), a total of 108 RTTs across the four participating sites were identified via screening and invited to complete the survey.

### Study outcome measures

#### Patient Participants

##### Demographics

At CT simulation, basic demographic information and baseline literacy assessment (see below) were collected from consenting participants. Demographical details pertained to age, gender, highest education level attained, number of years living in Australia, treatment bodily site, treatment facility location, and treatment fractionation.

##### Health and English literacy assessment

Consenting participants completed an assessment of English proficiency (LexTALE) and health literacy (REALM-SF) [12, 13]. The validated LexTALE assessment comprises sixty words, of which the participant is provided 5 min to ascertain which are true or false English terms. Analysis of these results allows for categorisation of English proficiency—given by the Common European Framework (CEF) [13]. The CEF offers a guideline to the proficiency of European languages. However, one must caution direct transferability in the English context.

Similarly, the validated REALM-SF poses seven health-related terms, to which the individual is scored on their ability to articulate the word. These tests served as a baseline measurement of each participant’s capability to communicate in English and/or understand health information. A resident interpreter (in-house or via agency) supported completion of these assessments.

##### Patient survey

A paper-based survey was completed anonymously by the patient at the end of the treatment course (see Appendix 1). The survey comprised ten questions, of which the first nine questions allowed for ‘Yes,’ ‘No,’ ‘Unsure,’ or ‘Not Applicable’ responses, with an optional free-text field. Question ten offered a free text field for any further comments. Administered in Chinese Simplified, free-text responses were back-translated to English for analysis. The study team opted not for Likert responses due to limited sample size and the likelihood of statistical insignificance. Free-text responses were thought to add detail and contextualise one’s reasoning in selecting an appropriate response.

#### RTT participants

##### Log sheet

A log sheet was completed by treating RTTs, outlining any issues encountered via free text fields. The log sheet captured details relating to treatment fraction, staff members present, device used (Y/N), technical issues experienced (Y/N), and a free text field for optional comments.

##### Staff survey

Following the cessation of the study, all RTT staff engaged in the clinical use of the device were invited to participate in a short survey indicating their perceptions of the ITD (see Appendix 2). This survey was conducted via SurveyMonkey, and only participating RTTs were approached. Completion of the survey implied consent to the collection and use of the de-identified responses. All completed surveys were confidential and de-identified. Four questions were replicated across patient and RTT surveys—representing views on the ability to convey treatment instructions (Q4), engage in conversation (Q3), report technical issues (Q6), and overall satisfaction (Q7). These have been selected for inclusion within this report as they depict the key findings of the feasibility study.

### Statistical analysis

Statistical analysis drew upon quantitative data. All data was entered into a Microsoft® Office Excel 2003 spreadsheet (Version SP3, Microsoft ® Corporation, Redmond, USA) and descriptive statistics were used for primary data analysis. Qualitative data (provided in free text responses) were used to provide additional context in the responses provided and have been incorporated into this manuscript as direct quotations. Due to limited free text responses, thematic analysis was not possible.

## Results

### Participant demographics

#### Patient participants

Eighteen Mandarin-speaking participants consented to the study. One participant (ORBIT 15) did not complete the baseline assessment on account of staff oversight. A total of 7 participants failed to complete the post-treatment survey for reasons ranging from misplaced forms, reluctance to participate and/or administration error. Thus, the compliance rate with respect to returned patient surveys was 61.1%.

Females (*n* = 13) accounted for 72.2% of participants. Patient age ranged from 46 to 85 years, with a mean age of 62.6 years. Similarly, participants ranged in Australian residency from 6 months to 32 years, with a mean of 14.6 years. 41.2% of participants have attained tertiary qualification (TAFE/University, *n* = 7), 52.9% high school (*n* = 9), and 5.9% primary school (*n* = 1). The most common treatment type was breast/chest irradiation (44.4%, *n* = 8). Fractionation ranged from 5 to 39 treatments, with a mean of 19.5 fractions (~ 4 weeks). Moreover, 50% of participants were treated at Metro Site B (*n* = 9), 38.8% at Metro Site A (*n* = 7), and 5.6% at each of metro site C (*n* = 1) and regional site A (*n* = 1), respectively. Further details are outlined in Table 1.

**Table 1:** Patient demographics

Demographic	No. of participants (*n* = 18), *n* (%)
Age range	
40–49	3 (16.7%)
50–59	3 (16.7%)
60–69	7 (38.9%)
70 +	5 (27.8%)
Gender	
Male	5 (27.8%)
Female	13 (72.2%)
Years living in Australia	
0–10 years	5 (27.8%)
11–20 years	8 (44.4%)
20 + years	4 (22.2%)
Not stated	1 (5.6%)
Highest educational level attained	
Primary school	1 (5.6%)
High school	9 (50.0%)
Tertiary (TAFE)	3 (16.7%)
University	4 (22.2%)
Not stated	1 (5.6%)
Treatment site (body location)	
Breast/chest wall	8 (44.4%)
Brain/H&N	4 (22.2%)
Pelvis/prostate	3 (16.7%)
Lung	1 (5.6%)
Gastrointestinal tract	2 (11.1%)
No. of RT treatments (fractions)	
0–10 fractions	5 (27.8%)
11–20 fractions	7 (38.9%)
20 + fractions	6 (33.3%)

#### RTT participants

At the time of RTT survey distribution, fourteen RTTs were no longer employed at the service location or were on extended leave. Thus, 94 RTTs were eligible to complete the survey. 36 RTTs obliged, representing a response rate of 38.3%. RTTs working in metropolitan sites accounted for 80.5% (*n* = 29) of staff participants. A further 8.3% (*n* = 3) worked at Regional Site A, and 11.1% (*n* = 4) did not state the treatment site in which they worked. Further breakdown of patients and RTTs by their corresponding treatment location (facility) is given in Table 2.

**Table 2:** Patient and RTT participants by treatment location

Treatment facility	Study participants	
	Patients (*n* = 18) *n* (%)	Staff (*n* = 36) *n* (%)
Metropolitan site A	7 (38.9%)	17 (47.2%)
Metropolitan site B	9 (50.0%)	8 (22.2%)
Metropolitan site C	1 (5.6%)	3 (8.3%)
Metropolitan site D	0 (0.0)%	0 (0.0%)
Multiple metropolitan sites	0 (0.0)%	1 (2.8%)
Regional site A	1 (5.6%)	3 (8.3%)
Not stated	0 (0.0)%	4 (11.1%)

### English and health literacy scores

The results of the REALM-SF (Health literacy) and the LexTALE (English literacy) assessments are provided in Table 3 and 4, respectively. REALM-SF results indicated a majority (52.9%, *n* = 9) of patients presented with a zero score, indicating a health literacy level equivalent of a Grade 3 (US educational equivalent) or below, which constitutes the lowest level of health literacy comprehension. The REALM-SF indicates that this cohort would typically benefit from oral and/or audio materials. Similarly, all patient participants scored in the lowest domain of the LexTALE assessment (CEF Level B1 and below). The description of this domain indicates an English literacy at a level of ‘lower intermediate or below.’

**Table 3:** Baseline patient assessment (REALM-SF). Adopted from Arozullah et al. [12]

Health literacy (REALM-SF) results			
School grade range	Grade description	REALM-SF score	Patients (*n* = 17) *n* (%)
Year 9 or greater (secondary)	Will be able to read most patient education materials	7	2 (12%)
Year 7–8 (secondary)	Will struggle with most patient education materials—will not be offended by low-literacy materials	4 to 6	2 (12%)
Grade 4–6 (primary)	Will need low-literacy materials—may not be able to read prescription labels	1 to 3	4 (24%)
Grade 3 or lower (primary)	Will not be able to read most low-literacy materials—may benefit from repeated oral instructions, or materials comprised of illustrations, audio and/or video	0	9 (53%)

**Table 4:** Baseline patient assessment (LexTALE). Reproduced from Lemhöfer and Broersma [13], with permission granted under Creative Commons license, March 3rd 2024

English Literacy (LexTALE) Results			
Common European framework (CEF) level	CEF description	LexTALE score	Patients (*n* = 17) *n* (%)
C1 & C2	Upper & lower advanced/proficient	80–100%	0 (0%)
B2	Upper intermediate	60–80%	0 (0%)
B1 & below	Lower intermediate & below	Below 59%	17 (100%)

### Use of the ITD to convey treatment instructions (survey question 4)

**Fig. 1:**
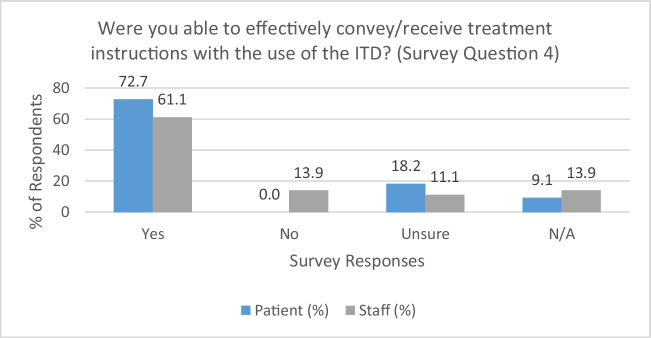
Ability to convey/receive treatment instructions

Both patients and RTTs reported affirmative results in the ability to convey and/or receive treatment instructions (see Fig. 1). 72.7% (*n* = 8) of patients and 61.1% (*n* = 22) of RTTs agreed that the ITD proved to be a reliable means of communicating critical treatment instructions to the patient to facilitate more effective daily treatment. Free text responses provided by RTTs suggested that the device was particularly useful in this setting—most notably with simple instructions, including directives for patient positioning. This is evidenced in comments provided by one RTT participant:

“*I felt this was the best use for the device. In conjunction with hand gestures, the patient was able to quickly understand what we were saying*”. (STAFF09)

### Use of the ITD to facilitate conversational language (survey question 3)

**Fig. 2:**
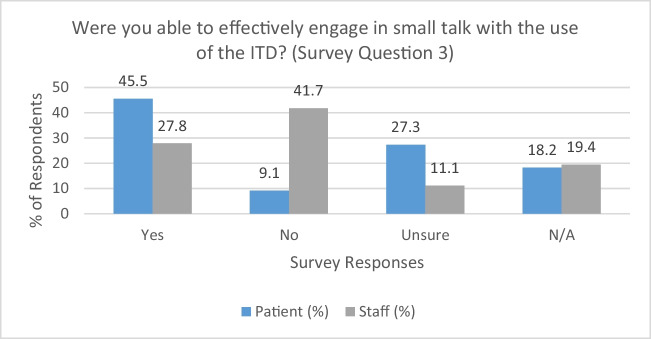
Ability to engage in conversation

There was a considerable disagreement of views from RTTs and patients when contemplating the role of an ITD in facilitating general conversation (see Fig. 2). 45.5% of patients (*n* = 5) felt that the ITD supported conversational communication, whereas the leading response of RTTs (41.7%, *n* = 15) was contrary to this notion. Akin to the points raised above, RTTs felt that the device was more appropriately used in providing instructions to the patient:

“RTTs *tended to use it more for getting important information across, rather than small talk*”. (STAFF08)

### Prevalence of technical issues (survey question 6)

**Fig. 3:**
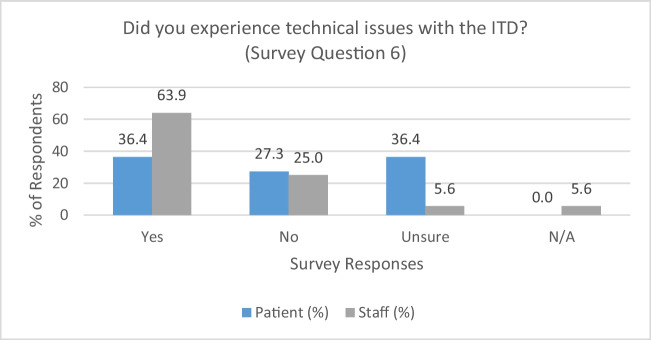
Prevalence of technical issues

Regarding the frequency of technical problems, differing opinions were observed between patients and RTTs. Figure 3 shows a strong inclination towards RTTs encountering technical issues (63.9%, *n* = 23). On the other hand, the patient data distributed across ‘yes,’ ‘no,’ and ‘unsure’ categories underline significant inconsistency and uncertainty within this group. Where indicated, the majority of RTTs and patients reported “*connectivity issues*” (ORBIT14) as the most common concern.

### Overall satisfaction (survey question 7)

**Fig. 4:**
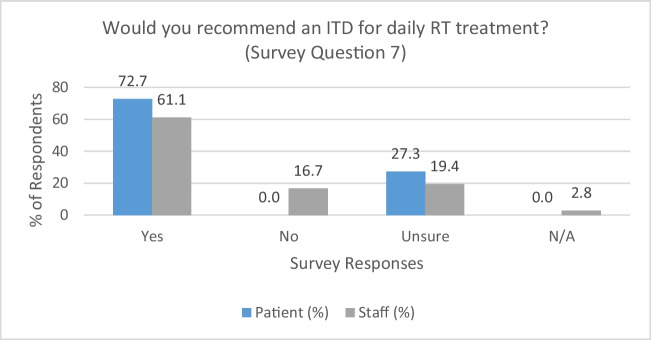
Recommending an ITD for daily RT treatment

Finally, the study results demonstrated parity in patient and RTT survey responses when asked if they would recommend the use of an ITD as part of daily RT treatment. As per Fig. 4, 72.7% (*n* = 8) of patients and 61.1% (*n* = 22) of RTTs, respectively, deemed that they would recommend the device. Qualitative feedback suggested the following:

“*using the device didn’t make the treatment feel ‘mechanical’”*. (ORBIT14),

“*it is helpful for those who do not speak English well*”. (ORBIT01), and

“*very practical*”. (STAFF14)

## Discussion

This feasibility study sought to facilitate daily engagement between Mandarin-speaking patients and RTTs with use of an ITD. To the knowledge of the authors, this presents the first healthcare application of a dedicated language translation device; thus, there are no known studies to draw direct comparison. Critical to the evaluation of this study was the assessment of clinical application—specifically the extent to which the ITD facilitated information provision and the ability to build rapport. The investigators utilized patient and RTT surveys to elicit perspectives on the suitability and acceptance of ITD use within daily RT service provision and viability for study expansion.

### Information provision and building rapport

This feasibility study demonstrated that a majority of patients and RTTs saw use for the ITD in conveying treatment instructions and information. An inability to communicate critical information between a health provider and a CALD cancer patient may otherwise limit active engagement, decision-making, and compliance throughout treatment [10, 14–18]. Supporting literature raises concern for the provision of information where there is an absence of a mutual language [10, 14–16].

In the RT context, treatment instructions may vary from ensuring patient preparedness in advance of attending for treatment (i.e., bladder filling, voiding bowel, and pre-medication requirements) to in-room treatment positioning requirements. Broader RTT information may encompass treatment scheduling, side-effect management, and self-care advice [19].

However, a disparity existed when patients and RTTs responded to the role the ITD played in facilitating informal conversation. Patient participants reported the device capable of striking a balance between information provision and building rapport. RTTs, however, saw less value in the use of the ITD for engaging in ‘small talk.’ Current literature reports a clear desire for CALD cancer patients to build trust and rapport with treating health providers, rather than merely facilitate a transactional engagement [17, 18]. This is best captured by Butow et al. (2011), who compare standard service provision to ‘… like being in a bubble, able to see, but unable to communicate with the outside world’ [17]. It is understood that improved engagement will help to address unmet emotional, coping, and support needs of CALD cancer patients [10, 16, 18]. Thus, it may be reasonable to associate the perceived value of the ITD in facilitating general conversation as relative to the disparate baseline (common) experience of healthcare by patient and staff participants, respectively.

### Suitability and acceptance of ITD use

This study revealed a higher rate of reported technical issues experienced by RTTs, as opposed to patients. Given RTTs facilitated the use of the device, these issues may not have been evident to the patient, should the device have been withdrawn from daily use, or rectified prior to clinical use. Signal drop out was commonly reported—addressed in part with a substitution from Wi-Fi to SIM connectivity. However, the nature and design of the bunker environment will continue to be a challenge for communications technology. As the study advanced, the use of SIM card connectivity increased in an effort to mitigate these issues, rather than relying on the hospital’s Wi-Fi.

In light of reported informal use of machine translation technology in the clinical domain, this study sought to determine clinical suitability and acceptance by patients and RTTs alike [10]. Prior research completed by the investigators had demonstrated confidence in the output accuracy for English to Mandarin translations and appropriateness with respect to technology and infection control protocols within the Victorian public RT service [11].

Despite varying perceptions of patients and RTTs on the scope of use and prevalence of technical issues, the use of the ITD within clinical practice was largely considered a positive addition to RT treatment provision.

### Study limitations and recommendations

In March 2020, the emergence of the COVID-19 pandemic drastically altered the provision of healthcare within Victoria [20–23]. To ensure public safety, restrictions were imposed on visitations, travel, contact time, and research activity across Victorian RT services [21, 22]. With concern for the timeliness of a return to usual care provision, the investigators elected to cease recruitment to the study with eighteen patient participants (90% of target).

Considering the current study recruitment, there is merit in expanding this research to draw upon a larger sample size of patients. This would allow for meaningful multi-variate analysis, such as the correlation of demographical features, health/English literacy, and frequency of device use to overall patient experience and satisfaction. The sample size employed in this study simply did not allow for any correlation with side-effect management, though this is intended for an expanded study. The expanded study design should seek to consider the impact of the device on patient care, treatment outcomes, and service delivery. Furthermore, scaled implementation of the current study design would present opportunity to evaluate and inform the requisite training and educational requirements for broad adoption.

In the interim, the authors would like to caution any recommendations for expanded use of these results across other healthcare disciplines and/or languages due to the small sample and scope of this feasibility study.

## Conclusion

This feasibility study sought to compare and contrast the perceptions of Mandarin-speaking patients and RTT participants, engaged with the use of an ITD for daily RT treatment. The outcomes indicated efficacy in communicating treatment instructions, but conflicting experiences when used to engage in everyday conversation. Moreover, while technical problems were reported unevenly, both patients and RTTs endorsed the use of the ITD within the RT setting.

This study demonstrated viability of the ITD within the context of the RT department. However, implementation was limited to a single public service within the state of Victoria. As such, study expansion would be required to satisfy statistical measures and inform both the impact of the device and requisite training requirements across multiple organizations/jurisdictions.

### Supplementary Information

Below is the link to the electronic supplementary material.

Supplementary file1 (DOCX 35 KB)
