# Supplementary material for: Piloting a language translation device for Mandarin-speaking patients presenting for radiotherapy treatment—assessing patient and radiation therapist perspectives
Source: Support Care Cancer. 2024 Mar 19;32(4):234. doi: 10.1007/s00520-024-08438-x (PMC10951040; doi:10.1007/s00520-024-08438-x)
Supplement: Supplementary file 1 — Supplementary file1 (DOCX 35 KB) [file 520_2024_8438_MOESM1_ESM.docx]

**Appendices**

***Appendix 1 – Patient Survey***

1. Do you believe that the instant translation device allowed you to communicate your treatment concerns with the radiation therapists?
2. Did you find that the translated conversation with your radiation therapists often made sense to you?
3. Were you able to engage in informal discussion (‘small talk’) with the radiation therapists?
4. Were you able to understand instructions provided to you during treatment by the radiation therapists?
5. Did you enjoy using the instant translation device?
6. Did you experience any technical issues with the use of the instant translation device?
7. Would you recommend the use of an instant translation device for daily radiation therapy treatment?
8. Did you feel supported and cared for by the radiation therapists?
9. Do you believe that the instant translation device provided a more positive experience at the radiation therapy centre?
10. If required, please provide any additional comments below:

Q1-9 allowed for 'Yes', 'No', 'Unsure' or 'Not Applicable' responses, plus a free-text field, 'Please explain your response'

Q10 simply offered a free text field and was considered optional.

***Appendix 2 – Staff Survey***

1. Did you find the instant translation to be a useful resource for daily communication?
2. Did you find that the instant translation device allowed you to obtain anticipated responses and actions from the patient?
3. Were you able to better engage in informal discussion (‘small talk’) with the patient?
4. Were you able to adequately convey instructions to the patient using the instant translation device?
5. Did you find that the instant translation device was easy to operate?
6. Did you experience any technical issues with the use of the instant translation device?
7. Would you recommend the use of an instant translation device for daily radiation therapy treatment?
8. Do you feel that the instant translation device allowed you to provide enhanced patient care to non-English speaking patients?
9. Do you believe that the instant translation device is a valuable resource for the radiation therapy department?
10. If required, please provide any additional comments below:

Q1-9 allowed for 'Yes', 'No', 'Unsure' or 'Not Applicable' responses, plus a free-text field, 'Please explain your response'

Q10 simply offered a free text field and was considered optional.
